# Smart Sensor Control and Monitoring of an Automated Cell Expansion Process

**DOI:** 10.3390/s23249676

**Published:** 2023-12-07

**Authors:** David F. Nettleton, Núria Marí-Buyé, Helena Marti-Soler, Joseph R. Egan, Simon Hort, David Horna, Miquel Costa, Elia Vallejo Benítez-Cano, Stephen Goldrick, Qasim A. Rafiq, Niels König, Robert H. Schmitt, Aldo R. Reyes

**Affiliations:** 1 IRIS Technology Solutions, 08940 Barcelona, Spain; 2 Aglaris Cell, 28760 Madrid, Spain; nuria@aglariscell.es (N.M.-B.);; 3 Department of Biochemical Engineering, University College London, London WC1E 6BT, UK; joseph.egan@ucl.ac.uk (J.R.E.); q.rafiq@ucl.ac.uk (Q.A.R.); 4 Fraunhofer Institute for Production Technology, 52074 Aachen, Germanyniels.koenig@ipt.fraunhofer.de (N.K.); r.schmitt@wzl.rwth-aachen.de (R.H.S.); 5 Aglaris Ltd., Stevenage SG1 2FX, UK; 6 Laboratory for Machine Tools and Production Engineering (WZL), RWTH Aachen University, 52074 Aachen, Germany

**Keywords:** smart sensors, cell manufacturing platform, cell expansion process, consensus

## Abstract

Immune therapy for cancer patients is a new and promising area that in the future may complement traditional chemotherapy. The cell expansion phase is a critical part of the process chain to produce a large number of high-quality, genetically modified immune cells from an initial sample from the patient. Smart sensors augment the ability of the control and monitoring system of the process to react in real-time to key control parameter variations, adapt to different patient profiles, and optimize the process. The aim of the current work is to develop and calibrate smart sensors for their deployment in a real bioreactor platform, with adaptive control and monitoring for diverse patient/donor cell profiles. A set of contrasting smart sensors has been implemented and tested on automated cell expansion batch runs, which incorporate advanced data-driven machine learning and statistical techniques to detect variations and disturbances of the key system features. Furthermore, a ‘consensus’ approach is applied to the six smart sensor alerts as a confidence factor which helps the human operator identify significant events that require attention. Initial results show that the smart sensors can effectively model and track the data generated by the Aglaris FACER bioreactor, anticipate events within a 30 min time window, and mitigate perturbations in order to optimize the key performance indicators of cell quantity and quality. In quantitative terms for event detection, the consensus for sensors across batch runs demonstrated good stability: the AI-based smart sensors (Fuzzy and Weighted Aggregation) gave 88% and 86% consensus, respectively, whereas the statistically based (Stability Detector and Bollinger) gave 25% and 42% consensus, respectively, the average consensus for all six being 65%. The different results reflect the different theoretical approaches. Finally, the consensus of batch runs across sensors gave even higher stability, ranging from 57% to 98% with an average consensus of 80%.

## 1. Introduction

In this paper, we describe the work related to smart sensors carried out as part of the EU Horizon 2020 project AIDPATH (Artificial Intelligence-driven, Decentralized Production for Advanced Therapies in the Hospital) for the control and monitoring task of the cell expansion process [1]. In AIDPATH, an adjustable AI-driven platform for automated Chimeric Antigen Receptor T cell (CAR-T cell) manufacturing is being developed containing a set of modular software and hardware tools whose objective is to provide equitable and affordable access to advanced therapies [2,3].

The incorporation of smart sensors facilitates the creation of sophisticated control strategies, providing a comprehensive understanding of the bioprocess and enabling more effective control and optimization. The disruptions in signals from hard sensors during cell culture can impact not only the process itself but also the loop control strategies in the bioreactors. These disturbances may be caused by factors such as environmental fluctuations, inherent biological system variability, or equipment malfunctions. Despite their significance, the user overseeing the system may struggle to determine whether these disturbances are temporary, within the expected range of variations, or indicative of a more significant issue that might require manual intervention.

Within the scope of this study, the AIDPATH platform, especially the FACER bioreactor, will benefit from the ability to identify disruptions in hard sensors caused by bubbles or other transient fluctuations. These disruptions have the potential to trigger unintended reactions, such as in the metabolite-dependent media feeding process. Recognizing these disruptions may play a crucial role in fine-tuning internal control strategies. Furthermore, the integration of smart sensors might be highly advantageous in notifying users of unexpected or unfamiliar situations that may require their attention. Thus, smart sensor implementation serves to establish an additional layer of control enabling a more resilient and optimized bioprocess.

In the last five years (2018–2023), advances in hardware, software, and sensor technologies have given rise to a diversity of applications and techniques applied to optimizing the control and monitoring of the cell culture process. For example, in [4] a survey was conducted of automated cell expansion trends and the outlook of critical technologies. Key performance indicators (KPIs) and attributes that are mentioned include foaming, cell count, cell viability, glycosylation of proteins, biomass, cell morphology, and size. With respect to key technologies, fluorescence and Raman spectroscopy are mentioned for the identification of molecular specificities. Furthermore, chemometrics, principal components analysis (PCA), and artificial neural networks (ANN) are mentioned as techniques to extract key information and patterns. On the other hand, [5] is a specific study of bioreactor automation driven by real-time sensing, in which a calibration curve is interpolated based on glucose, lactate, viable cell density, and total cell density. Wang et al. in [6] survey the development of novel bioreactor control systems based on smart sensors and actuators. The following sensors were mentioned: RFID (Radio Frequency Identification) based hydrogen gas detector, cell growth monitor, multiparameter sensor (hardware, e.g., pH, temperature, and dissolved oxygen (DO)), and a combination of optical density, DO, and pH. It is to be noted that current bioreactors specialized in the production of cell and gene therapies include less monitoring due to the nature of the product, associated regulation, and lack of technologies that allow real-time monitoring of the product. Typical bioreactors for advanced therapies provide readings for temperature, pH, and DO, whereas a smaller number read oxygen (O_2_) and carbon dioxide (CO_2_) in the exhaust line, and even fewer bioreactors are able to monitor glucose and lactate.

In [7], Reyes et al. conducted a more extensive survey of modern sensor tools and techniques for monitoring, controlling, and improving the cell culture process. Among the techniques identified are artificial neural networks to predict glutamine, glutamate, glucose, lactate, and viable cell concentrations; spectroscopy (near-infrared (NIR), mid-infrared (MIR), Raman, Florescence, …), optical sensors for O_2_, pH, CO_2_, free-floating wireless sensors, and PCA and partial least squares (PLS) regression to model viable cell density and antibody titers. Sensors are classified into seven main groups, as follows: **Data-driven sensors**: PLS, PCA, ANN, and Fuzzy logic, including rules to describe unknown state variables from known measurements. **Model-driven sensors**: Mass/energy balances, media composition vs. culture yield, kinetic equations, and thermodynamics. **Grey-box sensors**: Mechanistic and data-driven. Dynamic modeling, Kalman filter, and system linearization using Taylor series expansion. **Soft sensors**: Use non-invasive online spectroscopic methods such as NIR/MIR, 2D fluorescence, and Raman spectral data given the multidimensional complexity of the signal and the need for multivariate data analysis to relate the data to relevant process parameters. **Cascade control**: Involve two feedback controllers used to improve the dynamic response of the controllers by distributing the disturbance over a secondary loop where corrective measures are taken without affecting the primary loop. It is mentioned that this type of controller has been successfully applied in bioprocessing, particularly to control dissolved oxygen. **Model predictive control**: The controller response is based on a process model, which can be mechanistic, hybrid, or data-driven in origin. The model is capable of forecasting process events given process conditions and measurements from various input sensors. **Fuzzy control**: Transforms quantitative data into qualitative parameters by converting numerical data into a membership function, which is a value between 0 and 1 that defines the degree to which a certain variable fits a given fuzzy set. The values in the 0–1 scale are dependent on a predetermined knowledge of the range of possible values.

A survey of recent advances in soft sensors for the monitoring, control, and optimization of industrial processes can be found in the paper by Jiang et al. [8]. In the recent literature, one of the focuses of soft sensor technology is ‘deep learning’ which typically involves multi-layer neural networks [9,10,11,12,13]. In [9], Chai et al. apply deep learning to resolve missing data, whereas [11,12] deal with noise and uncertainty in data. In [13], Ha et al. categorize smart sensor systems as neural network-based vs. non-neural network-based, indicating typical non-neural techniques such as linear regression, principal components analysis, support vector machines, and random forest, among others. Smart sensors in the context of Industry 4.0 and the Internet of Things (IoT) are considered in [14,15], and in [14] Kalsoom et al. indicate that key features of industrial smart sensors include: low cost, data preprocessing, self-calibration, and self-diagnostics, among others. Finally, [16,17] consider data processing of time series in the context of outlier and anomaly detection for industrial applications.

The remainder of this paper is organized as follows: Section 2 explains and describes the infrastructure for the application of the smart sensors: the AIDPATH platform and the Aglaris FACER bioreactor; Section 3 provides details of the methods, experimental setup, datasets and smart sensor background, and development; Section 4 presents the results of applying the smart sensors to the batch run data, including validation of the alerts using a consensus approach; Section 5 provides a discussion section which reflects on relevant issues and considerations; finally, Section 6 presents the conclusions.

## 2. Background

In this section, the infrastructure for the application of the smart sensors is explained and described: firstly, the overall AIDPATH platform for AI-driven, automated CAR-T cell manufacturing, and secondly, the Aglaris FACER bioreactor for which the adaptive control and monitoring is developed.

### 2.1. The AIDPATH Platform: AI-Driven, Automated CAR-T Cell Manufacturing

The AIDPATH platform consists of two complementary modules: (i) manufacturing, and (ii) quality control, both of which are automated and centrally controlled by the COPE control software [18,19]. The manufacturing module includes devices for cell washing, selection, activation, electroporation, expansion, harvest, and formulation, with the perfusion-based Aglaris FACER bioreactor being a key element. The quality control module features devices to conduct analytics for cell quantity, viability, identity, and characterization. The use of tubing-kit-based devices and sterile connectors reduces the need for highly trained personnel, while the deployment of AI-supported control strategies in the expansion platform allows for feedback loops based on analytical measurements. The platform will ultimately be modular, manufacturer-independent, and allow for the parallel production of CAR-T cells. The control software COPE is specifically tailored to the current needs of digitalization in immune therapy and handles heterogeneous interfaces for different machines and devices using standardized communication protocols (e.g., OPC-UA). The software models the capabilities of the devices as services, enabling the creation and execution of flexible process protocols. In the case of the Aglaris FACER in the AIDPATH platform, this allows parameterizing and executing protocols for cell activation and expansion [19].

**Figure 1:**
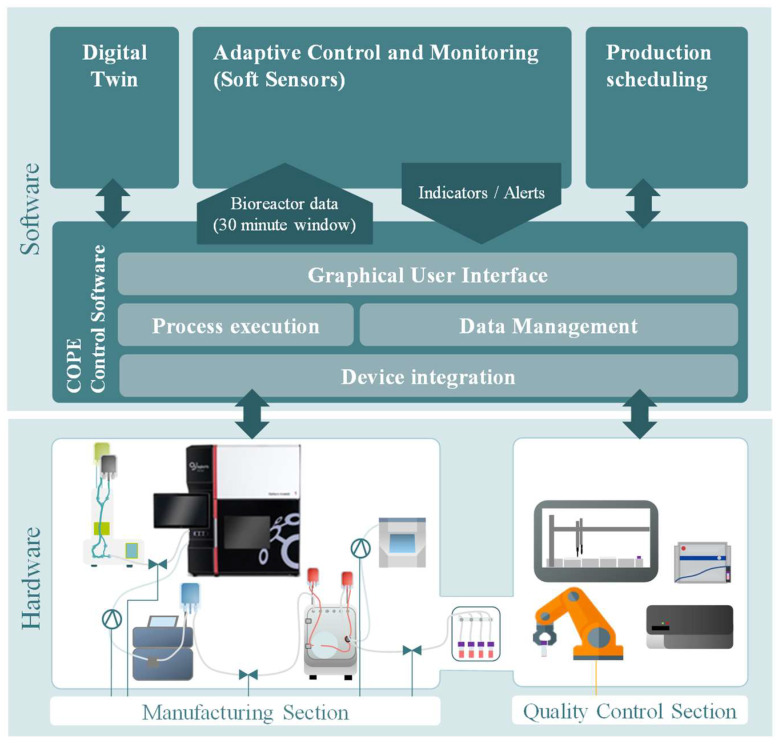
Architecture of automated control and monitoring of the Aglaris FACER on the AIDPATH platform.

With reference to Figure 1, in order to react adaptively to the cell behavior on the platform, the COPE’s data management system centralizes all data acquired from devices and sensors and makes them available to different modules and for analysis. Together with the Adaptive Control and Monitoring (ACM) smart sensor implementations described in this paper, a Digital Twin for adaptive production scheduling is also integrated and provided with the necessary input data. Their results are then fed back into the COPE and transferred to the platform. The COPE provides the relevant data from the Aglaris FACER sensors at thirty-minute intervals. The overall design anticipates that in the final stage, the Aglaris FACER can adjust some of its parameters according to the feedback from the Digital Twin and ACM. The overall concept is seen as an ‘alert system’ that provides actionable information on a dashboard, which the human operator can consult for decision support on possible adjustments or actions on the platform. These actions could imply an adjustment of the flow of media/gases and/or other parameters. Finally, this could lead to the prediction of the optimal cell harvest point which can then be used to adapt the production schedule.

As shown in Figure 1, the service-oriented architecture and semantically described interfaces will enable straightforward integration of modules. This architecture allows for a robust expansion process and, ultimately, a promising cancer therapy with broad patient access and reasonable costs.

The COPE interface acts as an intermediary between the Aglaris FACER hardware (lower left) and the Digital Twin and soft sensors (top left and center). The data buffer stores a 30 min sliding window of data which is generated at 10 s intervals from the expansion platform and is made available to the Digital Twin and soft sensors. The output from the Digital Twin (e.g., harvest point indicator) and soft sensors (e.g., stability indicators and alerts) are published in the data buffer and made available to the FACER via the COPE. In the current stage of the work, the COPE will provide simulated expansion data (from five historical batch runs in the FACER) and in a future stage the cell expansion hardware will be integrated. Note that in this paper, the terms ‘soft sensor’ and ‘smart sensor’ are considered synonymous.

### 2.2. Description of the Aglaris FACER Bioreactor

The Aglaris FACER is a GMP-grade fully autonomous cell culture platform tailored to the needs of cell therapy. The equipment is designed to improve performance and mitigate contamination risks. The FACER operates autonomously, significantly reducing the need for human intervention. The cell culture is processed in a closed sterile single-use cartridge, as required for advanced cell therapy manufacturing, eliminating the need for cleaning and sterilization between batches.

A pivotal element in the FACER’s design is the integration of sterile single-use sensors within the cartridge. These sensors provide precise measurements of process parameters (mainly temperature, DO, pH, glucose, and lactate) while ensuring a sterile environment for each run by eliminating the manual interventions required for sampling. While temperature, DO, or pH single-use sensors are commonplace in various commercial bioreactors for cell and gene therapies [20,21,22], the measurement of high-frequency glucose and lactate concentrations in the culture is uncommon. Also, the possibility to closely monitor cell growth from these parameters is a unique feature of the FACER, to the best of our knowledge. Single-use sensor integration not only provides a comprehensive real-time characterization of the cell culture process, but also allows for rapid detection of deviations from optimal conditions (enabling prompt corrective actions), as well as real-time adjustment of certain culture parameters to the cellular needs. The incorporation of automation and process analytic technologies in cell therapy manufacturing provides a way to improve process robustness [23]. The combination of (1) full automation, (2) high sensing capacity, (3) ease of connectivity to other equipment, (4) the possibility of parallelized productions, and (5) versatility makes the Aglaris FACER an ideal expansion module for integration in the AIDPATH platform.

**Figure 2:**
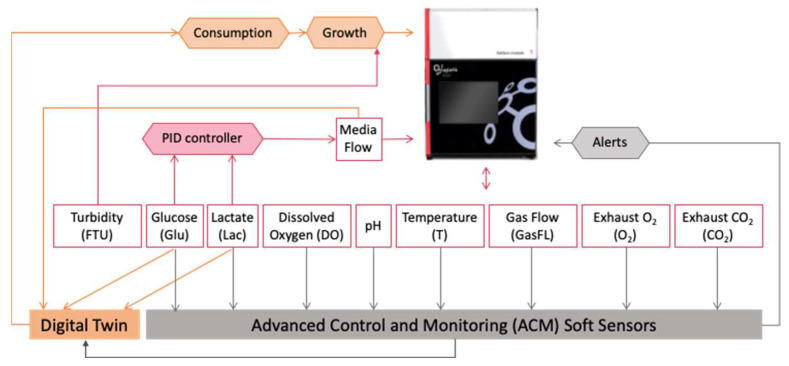
Aglaris FACER 2.0 sensing schematics.

Figure 2 shows a schematic diagram of the Aglaris FACER sensor array. It has different sensors to measure cell culture parameters: glucose (Glu) and lactate (Lac, enzymatic sensors), DO in medium (DO), medium pH, exhaust oxygen concentration (O_2_), exhaust carbon dioxide concentration (CO_2_), turbidity in the cell chamber (formazin turbidity units FTU, optical sensors), gas flow, and temperature (resistance temperature detector, T). Measurements of these sensors are collected by the Aglaris FACER’s control module to monitor the cell expansion process and optimize it. The Aglaris FACER correlates turbidity in the cell suspension chamber with the cell density. In order to control the media flow (perfusion) rates and adjust the feed to the culture at a given point in time, the Aglaris FACER makes use of a proportional-integral-derivative (PID) controller using feedback from the glucose/lactate enzymatic sensors. For the batch runs analyzed in this paper, only glucose readings were used as feedback for the PID perfusion rate control.

Within the AIDPATH environment, all the previously mentioned sensor measurements are shared with the ACM smart sensors (via the COPE) in order to detect certain events. In addition, these smart sensors will provide additional information that will be useful to support the operator’s evaluation of the process. Furthermore, glucose and lactate concentrations and perfusion rate will be sent to a Digital Twin model to predict cell growth and support the operator’s decision-making in order to choose the optimum point in time to harvest the cells for cryopreservation or reinfusion into the patient.

## 3. Methods

In this section, the methods, experimental setup, datasets, and smart sensor development are described. The batch run (FACER) datasets are first detailed (Section 3.1), followed by the smart sensor background and development (Section 3.2), the Weighted Weighted Average (*WWA*) smart sensor application to the FACER datasets (Section 3.3), and the Fuzzy smart sensor (Section 3.4).

### 3.1. Batch Run (FACER) Datasets

The datasets used for smart sensor development were captured from five expansions/batches of T cells or CAR T cells at clinically relevant scales conducted in the FACER. The general procedure used for cell production is described as follows: T cells were selected from peripheral blood mononuclear cells (PBMCs) and cryopreserved. After thawing, T cells were activated with TransAct™ (Miltenyi Biotec, Bergisch Gladbach, North Rhine-Westphalia, Germany) and expanded in the FACER in TexMACS™ medium combined with Human interleukin (IL)-7 and Human IL-15 (Miltenyi Biotec). Antibiotics were added in runs 1–4 but not in run 5. Human Serum was added in runs 1–3 and 5 where donor T cells were expanded but not in run 4 where patient CAR T cells (Clínica Universitaria Navarra, Pamplona, Spain) were expanded. Transduction in run 4 was performed with CAR lentivirus, provided by Clínica Universitaria Navarra. Each batch run used different initial conditions (quantity and donors/patients), and variable process conditions to provide different scenarios for smart sensor development and validation. During the expansion, sensor readings were polled at 10 s intervals.

Eight sensor measurements were pre-selected by the bioreactor process experts as the most significant for the cell expansion process, being those shown in Table 1.

For the validation, different combinations of the datasets were used to calibrate and then test the smart sensors. For example, a first calibration was performed using batch runs 1 and 2 (more stable), then validation with batch runs 3 to 5 (less stable). Then, a second calibration was performed using batch runs 3 to 5, with validation on batch runs 1 and 2. In this way, thresholds were found that were optimal and a trade-off for all five batch runs.

Regarding the nature of the datasets, batch runs 1 and 2 were quite stable runs that output the expected cell production, and batch run 3 is an example of a biologically successful run that experimented with a mechanical issue mid-culture which stopped perfusion and affected the monitoring and sensing. Hence, batch run 3 can be used to test whether the smart sensors are capable of detecting, and maybe in the future, minimizing the effect of this kind of event. Batch runs 4 and 5 are successful runs with some instabilities that should be detected by the smart sensors. For all datasets, an initial unstable phase is expected when all parameters are slowly stabilizing around the set conditions, followed by a more stable phase.

**Figure 3:**
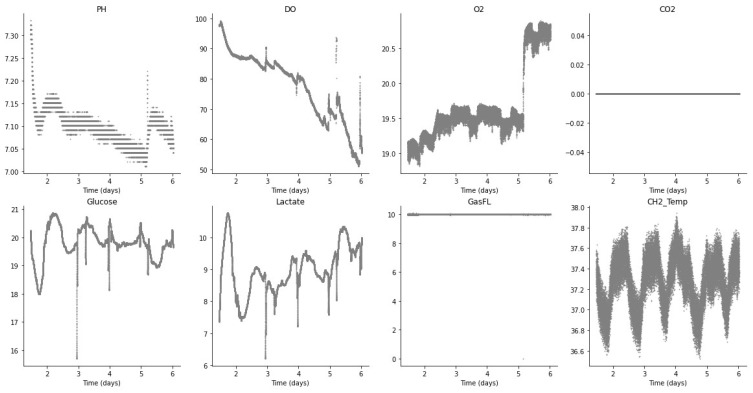
Sensor readings (batch run 2).

For control and monitoring, it is important to identify anomalous or non-optimal conditions and to provide a stable signal (without non-biological disturbances) as a guide for the PID controller. From the sensor data value distributions in Figure 3 (batch run 2), it can be seen that some distributions have multiple peaks, and values are also dependent on the corresponding time period during cell expansion, as well as the setpoints defined during the process.

Figure 3 shows the plots for each of the sensors during batch run 2, with time in days represented on the *x*-axis and the sensor value on the *y*-axis. It can be seen that for batch run 2, the CO_2_ sensor was inactive and the GasFL sensor value was constant. On the other hand, the glucose and lactate sensor values show a characteristic trend that is related to the evolution of the cell expansion perfusion process. Also, the DO sensor shows a progressive decrease whereas the temperature sensor value oscillates through the batch run (however, only through a small absolute range as shown on the *y*-axis).

**Figure 4:**
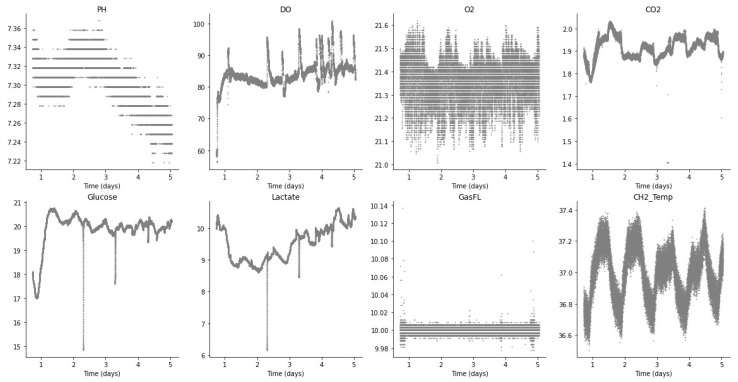
Sensor readings (batch run 1).

Figure 4 shows the plots for each of the sensors during batch run 1, which is representative of other productions in the Aglaris FACER using the same perfusion protocol. After the initial pre-stabilization stage, it can be observed that glucose tends to stabilize towards its setpoint, while lactate increases slightly over time. On the other hand, pH decreases compared to the values in the fresh media and DO displays some oscillation with respect to its mean value. O_2_ and CO_2_ concentrations in the gas phase remain stable (with small-scale oscillations) around their setpoints, with temperature and GasFL behaving in a similar manner.

**Figure 5:**
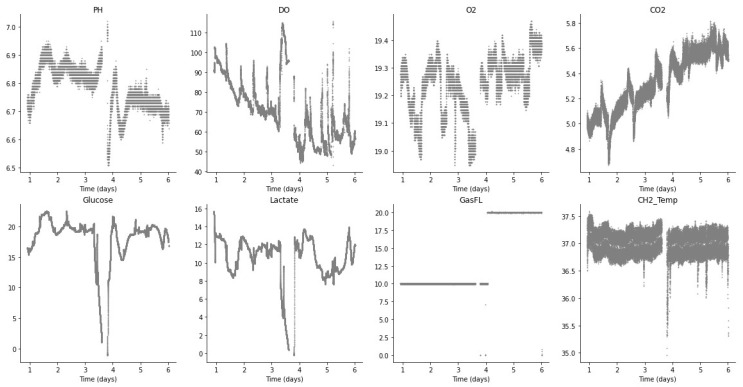
Sensor readings (batch run 3).

Figure 5 shows the plots for each of the sensors during batch run 3, in which there was a mechanical shut-down in the middle of the batch run (between days 3 and 4). However, the system was able to restabilize and complete the run with acceptable cell count and quality. The steep drop and recovery of the glucose and lactate values can be seen in a short period when the system was in shut-down and the sensors were not active. From day 4 onwards, setpoint readjustments to compensate for the shutdown have more atypical values and trends (e.g., CO_2_, GasFL) with respect to normal runs 1 and 2 (refer to Figure 3 and Figure 4).

### 3.2. Smart Sensor Background and Development

**Figure 6:**
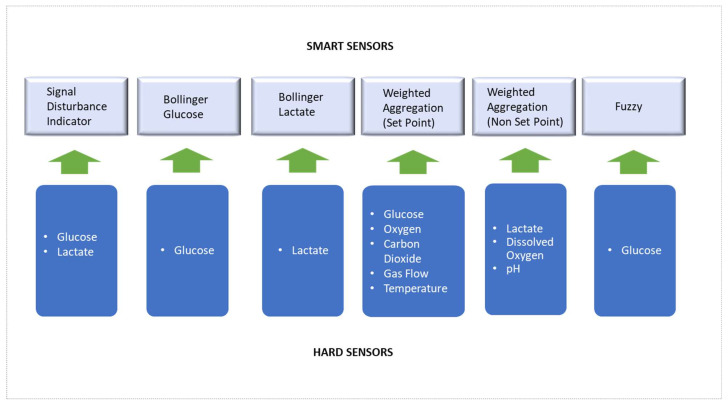
Relation of hard sensor inputs to smart sensors.

The following gives the background and theoretical basis of the techniques used for the smart sensors developed in this work, and how they are applied to the control and monitoring of the Aglaris FACER. The smart sensor techniques are Signal Disturbance Indicator, Bollinger Bands, *WWA* aggregation, and Fuzzy controller, with the corresponding hard sensor inputs as depicted in Figure 6. Finally, the consensus approach is detailed for polling the different techniques to obtain an overall decision support recommendation for the platform’s control and monitoring.

#### 3.2.1. Signal Disturbance Indicator

The Signal Disturbance Indicator (SDI) evaluates alterations in the signals and characterizes whether they are due to non-biological events. Some of these non-biological alterations could be a drop in the signal from glucose (accompanied by a drop in the lactate signal) due to a sudden change in temperature (i.e., the user opening the door to perform samplings), and/or transitory bubbles in the sensor array. Any of these disturbances, not due to cell behavior, might alter the signal trends (glucose, lactate, DO, pH) and the PID response may result in inaccuracy at those timepoints. Evaluating these events and identifying if they are related to cell behavior or not is valuable information that can be looped back into the system in order to correct and mitigate undesired effects.

Although the Aglaris FACER has temperature and bubble sensors in different parts of the circuit to measure these events, smart sensors can become a key tool to add extra valuable information to those measurements.

The developed SDI uses simple mean and standard deviation together with covariance in order to detect signal disturbances (i.e., a significant simultaneous drop in both glucose and lactate readings over a relatively short period of time). The cut-offs are established by statistical analysis of the available historical data, taking into consideration the standard deviation (σ) of the previous 30 min time window for the glucose (*G*) and lactate (*L*) trends over time. In particular, an event is defined as a disturbance if:

(1) $$G_{i}\;<\;\left(\;G\;\pm \;\sigma \left(G\right)\times \kappa \right)\;\;$$

and

(2) $$L_{i}\;<\;\left(L\;\pm \;\sigma \left(L\right)\times \kappa \right)\;\;$$

where *i* = 1:number of timepoints and κ is a constant varying from 2 to 4 in order to estimate the magnitude of the disturbance’s effect (small, medium, and large), respectively.

#### 3.2.2. Bollinger Bands [24,25]

Bollinger Bands (BB) consist of an N-period (time window of N) moving average (*MA*), an upper band at *K* times an N-period standard deviation above the moving average (*MA* + σ*K*), and a lower band at *K* times an N-period standard deviation below the moving average (*MA* − σ*K*). The resulting plot can thus embody stochastic/kinetic behavior and/or assumptions of the systemic setup and is not limited to the raw data value per se. The Bollinger band is typically used for tracking time series in financial data (stocks, etc.) but has also been successfully used for engineering applications. Thus, at point *i*:

(3) $$B{B_{i}}_{u}\;=\;MA_{i}\;+\;\sigma_{i}\;\times K$$

gives the upper band, and:

(4) $$B{B_{i}}_{l}\;=\;MA_{i}\;-\;\sigma_{i}\;\times K$$

gives the lower band.

For the bioreactor data (runs 1 to 5) the K value was calibrated to K = 8.

Next, the width of the range (from lower to upper band) was calculated:

(5) $$WR\;=\;B{B_{i}}_{u}\;-\;B{B_{i}}_{l}$$

In addition, the distance $\;D{V_{i}}_{u}\;,\;D{V_{i}}_{l}$ of data value *i* ($DV_{i}$) from each bound:

(6) $$D{V_{i}}_{u}\;=\;B{B_{i}}_{u}\;-\;DV_{i}$$

(7) $$D{V_{i}}_{l}\;=DV_{i}\;-\;B{B_{i}}_{l}$$

We take the smallest distance:

(8) $$DV\;=min(\;D{V_{i}}_{u}\;-D{V_{i}}_{l})$$

Obtain the distance as a percentage:

(9) $$DV_{p}=\frac{DV}{WR}\times 100$$

The alert threshold AT was assigned as 15% in the case of glucose and 20% for lactate. This was set together with the bioreactor expert by studying the results of processing data from different batch runs. Hence, if the distance percentage $DV_{p}$ becomes less than or equal to the threshold AT, the alert is triggered (assigned as 1); otherwise, the alert is assigned as zero, thus:

(10) $$DV_{p}\leq AT\to Alert=1$$

(11) $$DV_{p}>AT\to Alert=0$$

With reference to the application of Bollinger band sensors to the FACER control and monitoring, the following observations are made:

- If the current sensor value becomes too close to the upper or lower bound, this can trigger an action/alert.
- Trends can be identified—in general, the bands should reduce/converge towards the mean during the perfusion stage. The mean value for glucose should stabilize, which is considered a positive evolution given that a key control criterion is to keep the glucose level stable.

**Figure 7:**
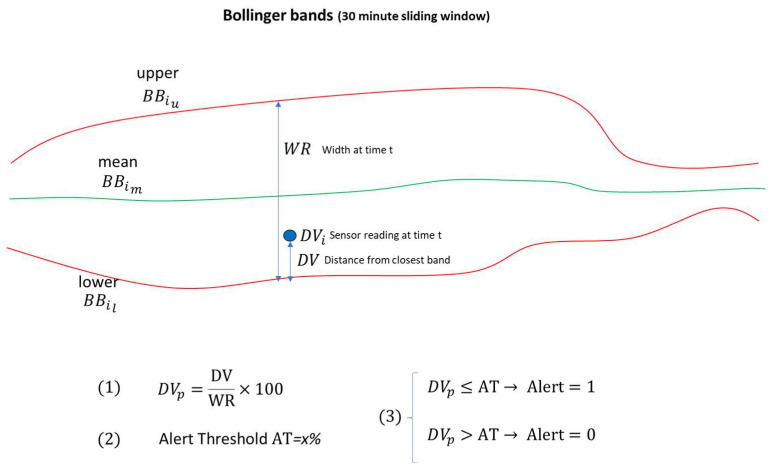
Concept of Bollinger Bands for Glucose and Lactate Monitoring.

The overall concept of the Bollinger Band Smart Sensor for Glucose and Lactate monitoring is illustrated further in Figure 7.

#### 3.2.3. WWA—Weighted Weighted Average

*WWA* represents an adaptation of the WOWA (Weighted Ordered Weighted Average) aggregation operator [26,27,28]. *WWA* is a versatile data aggregation and weighting technique, which on the one hand provides a scaling weight for variable values, and on the other hand, provides a critical range weight which represents the criticality of variations in the data values of each variable.

(12) $$WWA\;=\;\frac{\sum_{i=1}^{n}s_{i}\times \;v_{i}\;\times w_{i}}{n}$$

where:

*s_i_* is the distance from the set point or reference value of the data value of sensor *i*.
*v_i_* is the scaling weight of sensor *i*.
*w_i_* is the critical range weight, representing the criticality of distance from the set point or reference value of sensor *i* (*s_i_*).
(e.g., for glucose, if *s_i_* < 0.8, then *w_i_* = 0; if *s_i_* ≥ 0.8, then *w_i_* = 1.
*n* is the number of sensors.

As well as the data rows, the input to the *WWA* includes two vectors (scaling and range criticality) which are used to weight and merge the data in a flexible and customizable way.

The *WWA* is particularly useful for sensors and sensor data, where it is not desirable to entirely exclude variables/data values. *WWA* instead dampens or potentiates them using the weights depending on their relative scaling and critical range. One challenge is the correct assignment of the weights, which can be completed by a combination of statistical evaluation and domain expert knowledge and insights. The scaling weight of each sensor variable (defined by the process experts) can be interpreted as their impact on overall system behavior, control, and outcome. The critical range weight uses a threshold (also defined by the process experts) for each sensor: if the sensor value is below the threshold, the criticality weight is 0; if the sensor value is equal to or above the threshold, the criticality weight is 1. The weights and thresholds were validated with the bioreactor expert by observing the *WWA* outputs for different historical batch runs.

#### 3.2.4. Fuzzy Controller [29,30,31,32]

Fuzzy control does not necessarily require any initial knowledge of system dynamics. It transforms quantitative data into qualitative parameters by converting numerical data (such as glucose sensor data range) into a membership value for different fuzzy sets, giving a value between 0 and 1 that defines the degree to which a certain variable fits a given fuzzy set. The values in the 0–1 scale are dependent on a predetermined knowledge of the range of possible values.

Fuzzy controllers are based on fuzzy set theory which have an advantage with respect to other deterministic and classifier systems: they allow membership of a data record to more than one class, each with its ‘grade of membership.’ As an example, consider the distance of the glucose level from the glucose sensor set point (as was explained for the *WWA* smart sensor). This distance can belong to two different fuzzy sets: ‘normal’ and ‘alert.’ For a given glucose level, the distance from the set point could belong to ‘normal’ with a membership value of 0.75 and to ‘alert’ with a membership value of 0.25.

Fuzzy sets are those whose elements have degrees of membership. Fuzzy sets were introduced by Zadeh in 1965 as an extension of the classical notion of set. Formally, a fuzzy set is a pair (U,m) where U is a non-empty set and m:U→[0,1] is a membership function. The reference set U is called the universe of discourse, and for each x∈U, the value m(x) is called the grade of membership of x in (U,m). The function $m=m_{A}$ is called the membership function of the fuzzy set A=(U,m).

For a finite set $U=\{x_{1},\ldots ,x_{n}\}$, the fuzzy set (U,m) can be denoted by $\{m(x_{1})/x_{1},\ldots ,m(x_{n})/x_{n}\}.$

Let x∈U. Then, x is called

- not included in the fuzzy set (U,m) if $m\left(x\right)=0$ (not a member);
- fully included if $m\left(x\right)=1$ (full member);
- partially included if $0<m\left(x\right)<1$ (fuzzy member).

The non-fuzzy (crisp) set of all fuzzy sets in a universe U is denoted with SF(U).

#### 3.2.5. Consensus

Four different paradigms are applied from statistics (SDI, Bollinger) and artificial intelligence (*WWA*, Fuzzy) with a similar objective, which is to measure and quantify the ‘stability’ of the system. This makes it possible to establish a ‘consensus’ approach which asks each technique and smart sensor for a stability evaluation, and then compares the similarity/difference of the replies and applies a function to show the consensus. The consensus can be based on, for example, the number of smart sensors giving an alert at the same time, so if 3 or more of the smart sensors indicate an alert, according to the previously calibrated thresholds, then the human operator can be recommended to pay special attention to the situation.

Applying consensus theory is a good approach for critical systems, as it avoids overfitting on any one technique, and increases the confidence factor of an overall alert/recommendation to the human operator of a control and monitoring system.

### 3.3. WWA Smart Sensor Applied to the FACER Datasets

The *WWA* includes the intrinsic information value of the descriptive data in terms of scaling of the sensor value and range criticality of the data value. To develop the *WWA* smart sensor, the pre-selected sensors were segregated into 2 categories: sensors with setpoints (SP) and sensors without setpoints (NSP). In sensors with a setpoint, the sensor reading should approximate the sensor setpoint. For the rest, no sensor setpoint is defined, but a probable range for the sensor values during perfusion was defined for each sensor based on historical data. The average point is the reference point used in the *WWA* smart sensor. A summary of the set points and reference values for the eight sensors is given in Table 2. It can be seen that the set point for glucose was 20 (mM) for batch runs 1 to 4 and 19 (mM) for batch run 5.

For the *WWA* to add extra value to the signals in the system, we make use of weighting and probabilistic values. For these datasets, the weights which are shown in Table 3, depend on the parameters and conditions initially selected (Table 2 and Table 3).

Table 3 shows the *scaling weight*, *critical range distance,* and *critical range weight* assignments to the selected sensors. For a given sensor, if the distance of the sensor value from the set point (Table 2) is greater than or equal to the criticality distance (Table 3), then the criticality weight will be 1 (which indicates ‘alert’), otherwise it is 0 (indicating ‘normal’). This approach is used for the *WWA* aggregation function smart sensor.

The first weighting vector refers to the scaling of a sensor value with respect to the values of other sensors. The scaling of a sensor value is considered static, i.e., it does not vary during a batch run or for different batch runs (as long as the production conditions remain the same, i.e., type of media, perfusion mode, and range of cell number). In Table 3, it can be seen that the scaling weight is 1 for O_2_, CO_2_, T, GasFL, lactate, and pH. On the other hand, a scaling weight of 0.5 is applied to glucose and DO. This is because the values of glucose and DO are relatively greater than the other sensors, hence the weight value of 0.5 reduces their effective values for input to the aggregation operator.

The second weighting vector applied in the *WWA* aggregator function considers the criticality of data values of each sensor, and it is therefore dynamic, dependent on the sensor value at each time interval. 

From Table 3, it can be seen that the criticality distance is in general different for each sensor, and as mentioned previously the criticality weight can have a value of 0 or 1 depending on whether the sensor value distance from the set point is within the critical distance or not.

### 3.4. Fuzzy Smart Sensor

**Figure 8:**
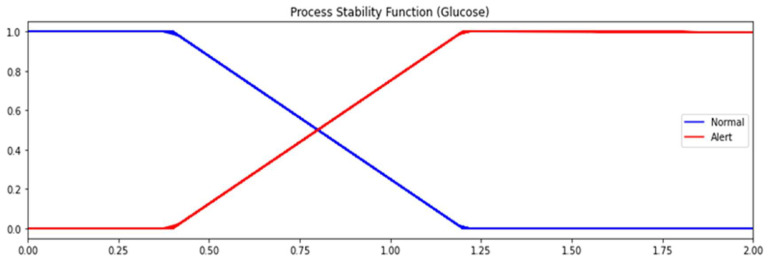
Fuzzy set representation for process stability (Glucose).

The fuzzy controller allows multiple grades of membership to different states, which may occur in ambiguous situations or noisy processes. Figure 8 shows the definition for the glucose sensor (represented by the absolute difference of glucose value to glucose setpoint) is represented by two fuzzy sets, ‘normal’ and ‘alert’. ‘Normal’ ranges from 0 to 1.2 and ‘alert’ ranges from 0.4 to infinity. This provides an alert with the grade of membership based on the distance of the glucose value from the setpoint. For example, with reference to Figure 8, if the distance from the setpoint is 0.8 (*x*-axis value), the grade of membership to ‘normal’ will be 0.5, and the grade of membership to ‘alert’ will be 0.5. If the distance from the setpoint is 1.0, the respective grades of membership to ‘normal’ and ‘alert’ will be 0.25 and 0.75, and if the distance from the setpoint is 1.5, the grades of membership will be 0.0 and 1.0, respectively.

Finally, in summary of the definitions of the smart sensors in Section 3, Table 4 shows the threshold values assigned for each smart sensor, calibrated from batch runs 1 to 5. If the smart sensor value is greater than the threshold, the alert value is 1, otherwise 0. The threshold values were calibrated by the bioreactor experts, evaluating the alerts produced in each batch run and then adjusting to correspond to the expected alerts.

## 4. Results

In this section, we show detailed use case examples of processing the data with the smart sensors for three of the five different batch runs of the Aglaris FACER for the cell expansion process. In Appendix A, the consensus plots are also given for batch runs 4 and 5. Each smart sensor approach (SDI, Bollinger bands, *WWA* daggregator, Fuzzy controller) provides additional information for the control and monitoring of the stability of the system. Firstly, Section 4.1 shows and comments on the plots of each smart sensor output and its corresponding alert; this is followed by Section 4.2, which shows a tabular summary of the consensus between the smart sensors for different batch runs and the correlation between different smart sensors, with an evaluation of the results.

### 4.1. Application of the Indicators and Smart Sensors to the Data

**Figure 9:**
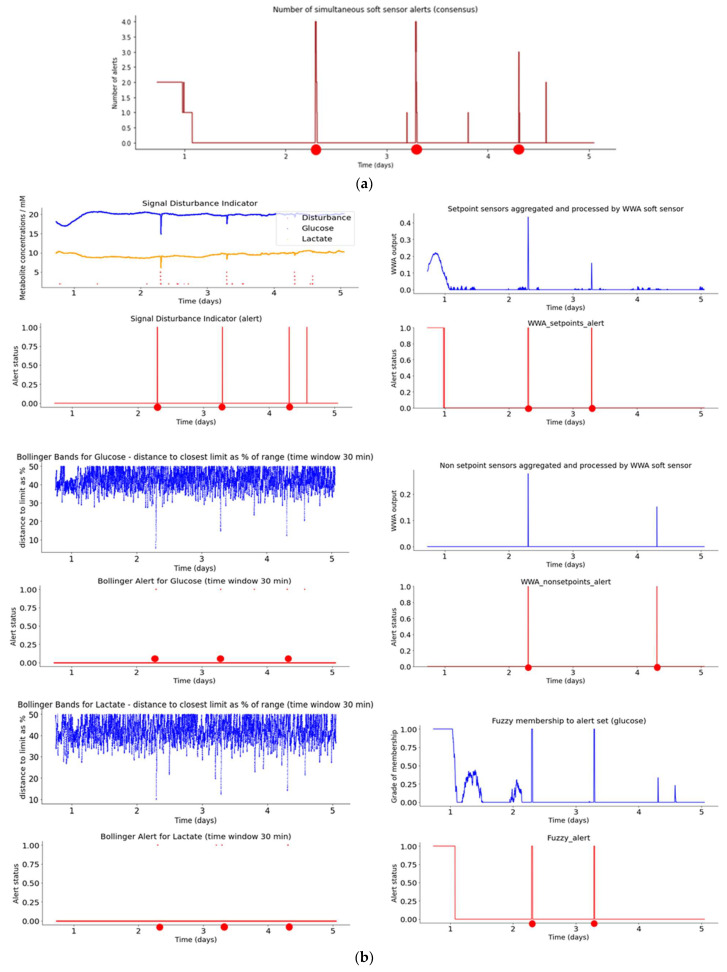
(**a**) Plot of number of simultaneous smart sensor alerts (consensus) during bioreactor run 1. (**b**) Plots for smart sensor outputs and alerts during bioreactor run 1.

For each batch run, first, a plot of the consensus function is shown for all the smart sensors, i.e., the number of simultaneous smart sensor alerts activated at each time interval during a given batch run. If the number is three or more, an event is identified (large red dot). Secondly, a plot is shown of the output of each smart sensor together with its corresponding alert status. The events identified on the consensus plot are then superimposed on each individual alert status plot and the ones which coincide are identified. For example, in Figure 9a three events are identified when three or more sensors are activated at the same time. Then, in Figure 9b, these three events are contrasted with the individual alert plots—for the SDI, Bollinger_Glucose, and Bollinger_Lactate smart sensors, the alerts coincide with all three consensus events of Figure 9a, whereas for *WWA*_NP, *WWA*_NSP, and Fuzzy, the alerts coincide with two out of three events of Figure 9a.

Hence, Figure 9a shows the number of simultaneous smart sensor alerts (consensus) during bioreactor run 1. It can be seen that three or more alerts are activated at three points (red dots at 2.3, 3.3, and 4.3 days). This plot supplies key information for decision-making regarding potential adjustments to the bioreactor control by the human operator.

Figure 9b illustrates the plots for Aglaris FACER run 1 of the indicators and smart sensors, with the red dots indicating ‘alert’ events. Figure 9b (top to bottom, left to right) shows the plots of the SDI and the Bollinger bands (glucose and lactate), the *WWA* aggregator for setpoint sensors, non-setpoint sensors, and the fuzzy sensor. For each smart sensor, the first plot is the output value of the smart sensor itself and the second plot is the alert status (1 or 0) depending on the threshold applied (see Table 4). So, for example, the SDI has a threshold of three bubbles (small dots rising vertically from the *x*-axis) for the alert to activate, which coincides on four points with the consensus (Figure 9a) during the batch run. For the Fuzzy sensor, the alert is activated when the grade of membership to fuzzy set ‘alert’ goes above the threshold of 0.45 (Table 4), which coincides also on three points (large red dots) with the consensus (Figure 9a). In general, the indicators show a relatively stable batch run with few alert events. 

Note that the *x*-axis scale is similar for all plots (units = days) whereas the *y*-axis scale is dependent on the sensor values and the metric.

**Figure 10:**
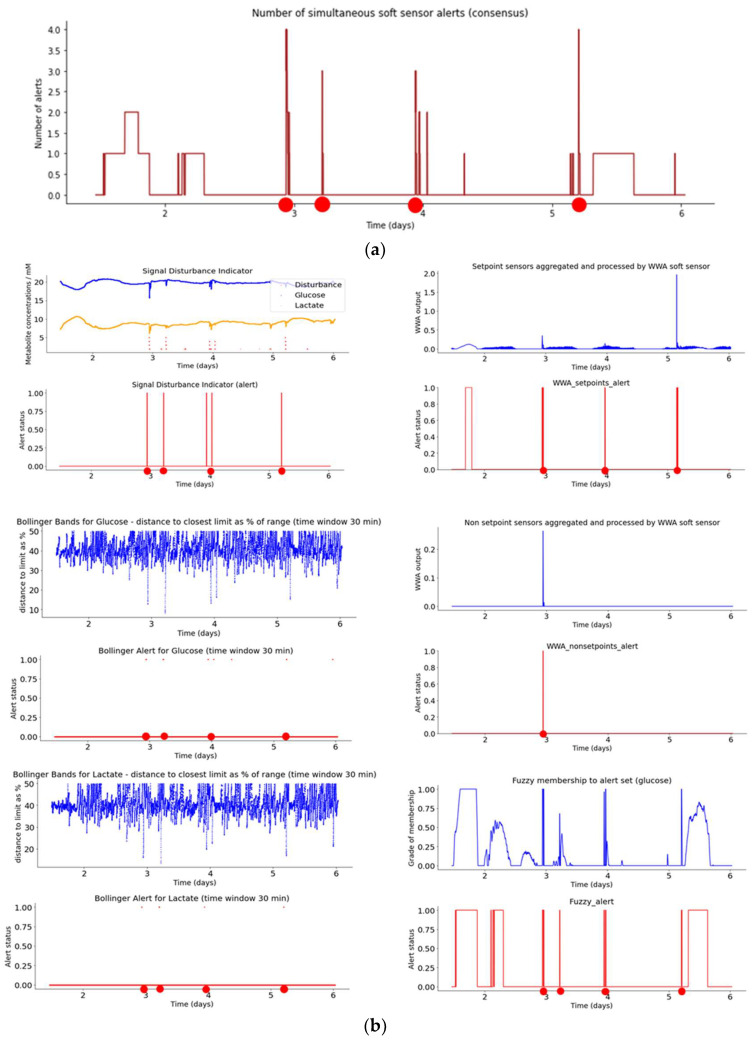
(**a**) Plot of number of simultaneous smart sensor alerts (consensus) during bioreactor run 2. (**b**) Plots for smart sensor outputs and alerts during bioreactor run 2.

Figure 10a shows the number of simultaneous smart sensor alerts (consensus) during bioreactor run 2. It can be seen that consensus events are indicated when three or more sensors give an alert at the same time, which occurs in four points (red dots at 2.9, 3.25, 3.9, and 5.25 days). Hence, comparing Figure 10a with Figure 9a, run 2 displays one more alert event than run 1. This plot again supplies important information to the human operator as support to decision-making regarding making any necessary adjustments to the bioreactor control points.

Figure 10b shows the indicator and smart sensor plots for Aglaris FACER run 2, following the same schema as for run 1 (Figure 9b). If we compare run 1 with run 2, it can be seen the trends are quite similar (especially glucose and lactate), with run 1 having one less alert event. In general, the indicators show a batch run that is slightly less stable than batch run 1, though with few alert events.

**Figure 11:**
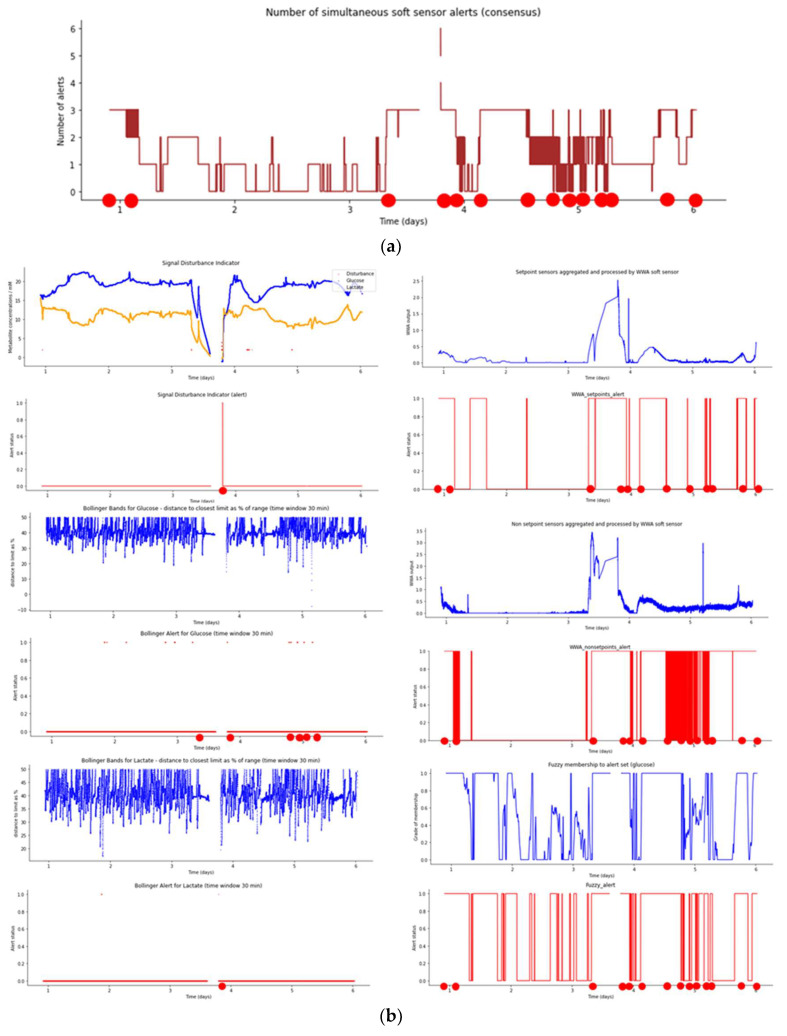
(**a**) Plot of number of simultaneous smart sensor alerts (consensus) during bioreactor run 3. (**b**) Plots for smart sensor outputs and alerts during bioreactor run 3.

Figure 11a shows the number of simultaneous smart sensor alerts (consensus) during bioreactor run 3. It can be seen that consensus events are indicated when three or more sensors give an alert at the same time, which occurs in fourteen points (red dots, mainly after the shutdown starting at approx. 3.5 days). Hence, it displays a significantly greater number of alert events than batch runs 1 and 2.

Figure 11b shows the indicator and smart sensor plots for Aglaris FACER run 3, following the same schema as for runs 1 and 2. In this run, perfusion was stopped (for about 8 h between days 3 and 4), and this is evidenced in the indicator and smart sensor plots. Days 4 to 6 show a good recovery of the system to a stable state; however, a greater instability (in comparison to runs 1 and 2) is still evident from the Fuzzy sensor. In contrast, the SDI shows a relatively smaller incidence of disturbances (red dots) before and after the perfusion stop.

For the consensus plots of batch runs 4 and 5, refer to Appendix A.

### 4.2. Consensus of the Smart Sensors

Table 5 shows the alert summary for batch runs 1 to 5, in which each smart sensor is compared to the “total consensus events” which represents the consensus, defined as points for which three or more smart sensors are activated simultaneously. Hence, the table captures how many consensus events a given smart sensor captured for a given batch run.

#### 4.2.1. Consensus with Respect to Smart Sensors

From Table 5, it can be seen that the SDI smart sensor picked up the least consensus events, with respect to other smart sensors, for batch runs 3 to 5. This is not necessarily a negative result, given that the SDI is looking for glucose and lactate signal disturbances in advance of the event which may not be aligned with other hard and smart sensor variations. Also, it can be seen that the *WWA*_SP (setpoint) smart sensor picked up the majority of events for all batch runs except the fourth, whereas the *WWA*_NSP (non-setpoint) smart sensor missed events for batch run 2.

In terms of the smart sensors, the rightmost column of Table 6 (%∑b) shows that Fuzzy (0.12) and *WWA*_NSP (0.14) had the most consensus over all batch runs, while SDI (0.75) and Boll_Lactate (0.58) had the least. Note that the larger the value of %∑b, the greater the difference between the number of events registered by a given smart sensor vs. the number of events registered as consensus events.

#### 4.2.2. Consensus with Respect to Batch Runs

From Table 6 (bottom row), it can be seen that there was the greatest consensus for batch runs 1 (0.02) and 2 (0.04) and the least for batch runs 5 (0.43) and 3 (0.3), where 0.02 represents the smallest percentage difference and 0.43 the greatest. For batch run 3, the perfusion issue can explain the difference with respect to other batch runs, whereas for batch runs 4 and 5, this is probably due to the different runtime conditions and bioreactor setup (glucose takes longer to stabilize and pH is below the expected values due to the large number of cells). Hence, future calibration will have to take further into account expected runtime conditions and setup.

Collectively, these results show that there was a reasonable overall consensus between the smart sensors for the different batch runs. However, it was concluded that the thresholds and weights will require further evaluation and calibration, as more batch runs under different conditions and more standard conditions become available. Furthermore, prototypic batch runs will be better defined for which we would expect fewer events and those for which we would expect more.

## 5. Discussion

With reference to Table 3, the scaling weights for each sensor were determined as follows. Initially, the idea was to have values between 0 and 1. However, during the calibration with the bioreactor experts, it was decided to assign 0.5 to the glucose and DO sensors and 1.0 to the other six sensors, as a value of 0.5 was found to scale down the values of glucose and DO with respect to the other six sensors. The key aspect is that the *WWA* aggregator smart sensor should produce an alert output that makes sense with respect to the batch run and the other sensors. In future work, the scaling weights could be further adjusted.

With respect to the five batch runs performed to evaluate the system and smart sensors, they were chosen and parameterized by the bioreactor experts and project coordinators within the design of the experiment as an initial calibration with mainly healthy patients. In the next steps of the project, more healthy donor batch runs will be added, and the system and the smart sensors will be further calibrated.

With respect to the calibration of the smart sensors across different batch runs (those that were stable versus those that were less so), this was completed in collaboration with the bioreactor experts who had actually performed the batch runs and had detailed knowledge of them. The adjustment of the alert thresholds should avoid too many alerts on the one hand, and insufficient alerts on the other, following the interpretation of the bioreactor experts for each batch run. More specifically, false positives (alerts without events) and false negatives (events without alerts) were scrutinized The aggregation smart sensors were more of a challenge given that they aggregate several hard sensors to provide one alert output, thus having a more complex behavior when compared to other smart sensors which depend on one or two hard sensors.

With respect to the unexpected shutdown and the ensuing re-stabilization in the third batch run, even though it was a mechanical issue, the Glu and Lac were decreasing progressively to 0, and this should be detected as a non-cellular behavior. Further calibration of the smart sensors and the consensus algorithm may be required to better detect a major event such as a mechanical shut-down. This could be completed by not only considering the number of simultaneous alerts but also the time the consensus is above three, for example.

Looking ahead to possible enhancements or new functionalities for the smart sensors to improve their detection and management of bioprocess instabilities, within the scope of the project new batch run data of healthy donors will be obtained, which will allow for further calibration so the system can generalize to as many potential patient cases as possible that can occur in a real hospital environment.

With respect to the manner in which the smart sensors inform decision-making during the cell expansion process in the Aglaris FACER bioreactor runs, the individual alert plots and consensus plot will be shown on the PLC controller dashboard. The human operators will consult the dashboard and evaluate this information together with existing monitoring and alert displays. As well as the number of simultaneous alerts at a given moment, the time a given number of alerts remain simultaneous is also a consideration. The time duration can be made more explicit in a future version of the consensus algorithm.

Reflecting on the presented results and observations, future adjustments can be recommended for the calibration of smart sensor thresholds and weights for upcoming batch runs. Particularly, this will be to adapt the alert thresholds to avoid too many alerts on the one hand and insufficient alerts on the other. The relevant events will be interpreted together with the bioreactor experts for each new batch run, and to assign values that also work for all previous batch runs. Furthermore, as well as adjusting the thresholds, a time factor (duration) will be introduced to determine when a consensus becomes an alert that needs user (human operator) interaction.

With respect to current limitations, the current batch runs are limited to five, and to mainly healthy patients; however, they have been carefully selected by the bioreactor experts as viable for initial calibration of the platform.

## 6. Conclusions

This paper has described six different smart sensors using four different AI and statistical paradigms, their calibration for a set of different bioreactor batch runs, and the results of applying the smart sensors to the batch run data captured from the hard sensors. A consensus approach has then been applied to serve as information to a human operator in order to suggest possible adjustments during the bioreactor batch run. This represents a complex control and monitoring application and shows how the smart sensors can provide an additional vision of the bioreactor monitoring to complement the current hard sensor data control information.

As the next steps within the ongoing AIDPATH project, the smart sensors will be (i) integrated into the overall IT platform including the control software COPE with the runtime interface to the bioreactor, and (ii) a dashboard will be developed to display the smart sensor outputs and alerts to the human operator in real-time. Further testing with new bioreactor runs will allow for further calibration of the smart sensors and adaptation as necessary.

## Figures and Tables

**Table 1:** Pre-selected sensors.

Parameter	Abbreviation	Units
Temperature	T	°C
Dissolved Oxygen	DO	% ais saturation (% a.s.)
pH	pH	-
Gas Flow	GasFL	mL/min
Glucose Concentration	Glu	mM
Lactate Concentration	Lac	mM
Exhaust Oxygen Concentration	O2	%
Exhaust Carbon Dioxide Concentration	CO2	%

**Table 2:** Sensor set points (SP) and reference values (RV).

Sensor	SP/RV	Batch Run1	Batch Run 2	Batch Run 3	Batch Run 4	Batch Run 5
Glu	SP	20	20	20	20	19
O_2_	SP	21	19.25	19.25	19.25	19.25
CO_2_	SP	1.9	5, 0	5	5, 1.9	5
T	SP	37	37	37	37	37
GasFL	SP	10	10	10	10	10
Lac	RV	10	10	10	10	10
pH	RV	7.1	7.1	7.1	7.1	7.1
DO	RV	72.5	72.5	72.5	72.5	72.5

**Table 3:** Sensor scaling weights, critical range distances, and criticality weights.

Sensor	Scaling Weight (*v_i_*) *	Critical Range Distance (*s_i_*) *	Critical Range Weight (*w_i_*) *
Glu	0.5	0.8	0 or 1
O_2_	1	0.5	0 or 1
CO_2_	1	0.5	0 or 1
T	1	0.5	0 or 1
GasFL	1	0.2	0 or 1
Lac	1	3	0 or 1
pH	1	0.35	0 or 1
DO	0.5	27.5	0 or 1

* Refer to Equation (12).

**Table 4:** Threshold values assigned for each smart sensor output.

Thresholds	
SDI	≥3 Bubbles
BB GLUC	25%
BB LACT	20%
*WWA* SP	0.1
*WWA* NSP	0.35
FUZZY	0.45

**Table 5:** Alert summary for Batch Runs 1 to 5.

	Batch Run				
	1	2	3	4	5
**Total consensus events**	3	5	14	14	21
**SDI**	3	5	1	5	0
**Boll_Glucose**	3	5	6	14	13
**Boll_Lactate**	3	4	1	12	4
***WWA*_SP**	2	5	12	7	16
***WWA*_NSP**	2	1	14	13	19
**Fuzzy**	2	5	14	8	21

**Table 6:** Consensus events undetected for smart sensors and batch runs.

	Batch Run						
	1	2	3	4	5	∑b	%∑b
**Total consensus events**	3	5	14	14	21	57	1
**SDI**	0	0	13	9	21	43	0.75
**Boll_Glucose**	0	0	8	0	8	16	0.28
**Boll_Lactate**	0	1	13	2	17	33	0.58
***WWA*_SP**	1	0	2	7	5	15	0.26
***WWA*_NSP**	1	4	0	1	2	8	0.14
**Fuzzy**	1	0	0	6	0	7	0.12
**∑s**	3	5	36	25	53	122	
**%∑s**	0.02	0.04	0.3	0.2	0.43		

∑b = sum for batch runs; ∑s = sum for smart sensors; %∑b = ∑b for each batch run divided by ∑b total consensus events; %∑s = ∑s for each batch run divided by ∑b total consensus events.

**Figure A1:**
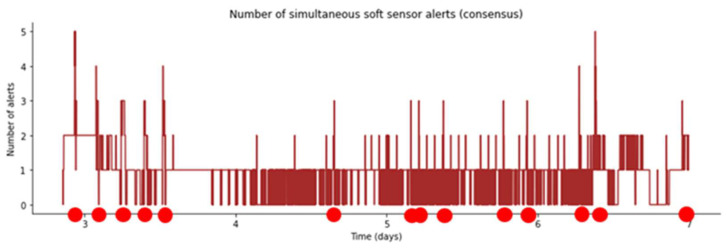
Plot of number of simultaneous smart sensor alerts (consensus) during bioreactor run 4 (red dots correspond to events with a consensus of 3 or more).

**Figure A2:**
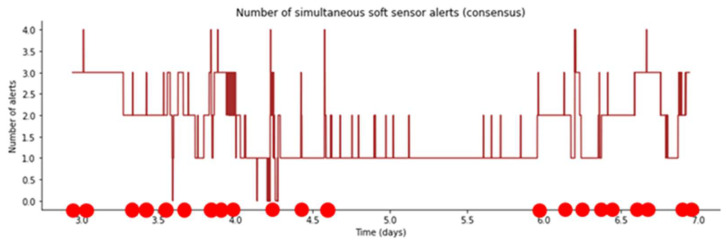
Plot of number of simultaneous smart sensor alerts (consensus) during bioreactor run 5. (red dots correspond to events with a consensus of 3 or more).

## Data Availability

The datasets presented in this article are not readily available because of the requirement to protect proprietary information. Requests to access the datasets should be directed to nuria@aglariscell.es.

## Appendix A

This appendix includes additional plots of the sensor data for batch-run datasets 4 and 5.
